# Global proteomic profiling of *Yersinia ruckeri* strains

**DOI:** 10.1186/s13567-017-0460-3

**Published:** 2017-09-20

**Authors:** Gokhlesh Kumar, Karin Hummel, Timothy J. Welch, Ebrahim Razzazi-Fazeli, Mansour El-Matbouli

**Affiliations:** 1 Clinical Division of Fish Medicine, University of Veterinary Medicine, Veterinärplatz 1, 1210 Vienna, Austria; 2 VetCore Facility for Research/Proteomics Unit, University of Veterinary Medicine, Vienna, Austria; 3 National Center for Cool and Cold Water Aquaculture, Kearneysville, USA

## Abstract

*Yersinia ruckeri* is the causative agent of enteric redmouth disease (ERM) of salmonids. There is little information regarding the proteomics of *Y. ruckeri.* Herein, we perform whole protein identification and quantification of biotype 1 and biotype 2 strains of *Y. ruckeri* grown under standard culture conditions using a shotgun proteomic approach. Proteins were extracted, digested and peptides were separated by a nano liquid chromatography system and analyzed with a high-resolution hybrid triple quadrupole time of flight mass spectrometer coupled via a nano ESI interface. SWATH-MS technology and sophisticated statistical analyses were used to identify proteome differences among virulent and avirulent strains. GO annotation, subcellular localization, virulence proteins and antibiotic resistance ontology were predicted using bioinformatic tools. A total of 1395 proteins were identified in the whole cell of *Y. ruckeri*. These included proteases, chaperones, cell division proteins, outer membrane proteins, lipoproteins, receptors, ion binding proteins, transporters and catalytic proteins. In virulent strains, a total of 16 proteins were upregulated including anti-sigma regulatory factor, arginine deiminase, phosphate-binding protein PstS and superoxide dismutase Cu–Zu. Additionally, several virulence proteins were predicted such as Clp and Lon pro-teases, TolB, PPIases, PstS, PhoP and LuxR family transcriptional regulators. These putative virulence proteins might be used for development of novel targets for treatment of ERM in fish. Our study represents one of the first global proteomic reference profiles of *Y. ruckeri* and this data can be accessed via ProteomeXchange with identifier PXD005439. These proteomic profiles elucidate proteomic mechanisms, pathogenicity, host-interactions, antibiotic resistance ontology and localization of *Y. ruckeri* proteins.

**Electronic supplementary material:**

The online version of this article (doi:10.1186/s13567-017-0460-3) contains supplementary material, which is available to authorized users.

## Introduction

Enteric redmouth disease (ERM) is one of the most important bacterial diseases of salmonids and causes significant economic losses in the aquaculture industry worldwide. ERM can affect fish from all age classes and appears as a more chronic condition in older and larger fish. The disease is caused by *Yersinia ruckeri*, a Gram-negative rod-shaped enterobacterium [1, 2]. *Y. ruckeri* enters the fish via the secondary gill lamellae and from there spreads to the blood and internal organs [3]. Clinical signs of the disease include exophthalmia, darkening of the skin in addition to subcutaneous hemorrhages in and around the mouth and throat. The spleen is often enlarged and can be almost black in color and the lower intestine can become reddened and filled with an opaque, yellowish fluid [1, 2]. Focal areas of necrosis can be present in the organs (spleen, kidney and liver). Degenerated renal tubules, glomerular nephritis and a marked increase in melano-macrophages may be observed in the kidney of infected fish [1, 2, 4]. Several virulence factors of *Y. ruckeri* have been identified such as extra-cellular products and Yrp1. Extra-cellular products have been shown to reproduce the clinical signs of the disease [5]. The 47 kDa metalloprotease Yrp1 is necessary for virulence and degrades fibronectin, actin and myosin of the fish [6].

Strains of *Y. ruckeri* have been categorized into two biotypes: biotype 1 strains are motile and lipase positive, while biotype 2 strains are negative for these phenotypes [2, 7]. Previously, the majority of epizootic outbreaks in salmonids were caused by biotype 1 strains which could be easily controlled by vaccination with a bacterin vaccine [5]. Nevertheless, biotype 2 strains have recently emerged and have been responsible for outbreaks in both naive and vaccinated fish, thereby suggesting that biotype 2 strains may be less sensitive to the traditional ERM vaccine which is made from a biotype 1 strain [8, 9]. This relationship between vaccine failure and emergence of biotype 2 has led to the hypothesis that the loss of the flagellum is essential for resistance to immersion vaccination [9, 10]. However, bivalent or biotype 2 vaccines provide good protection against the biotype 2 strains [2, 11].

Whole genome sequences of *Y. ruckeri* strains have been annotated and can now be used for comparative genomic analysis of strains and other research purposes [12]. Global proteomic identification and comparative analysis of *Y. ruckeri* strains are required to create a proteomic map, understanding proteomic biology, proteomic changes and proteomic differences between strains. Little is known about the proteomics of *Y. ruckeri*. Outer membrane protein and whole cell protein patterns of *Y. ruckeri* isolates were described using SDS-PAGE and 2D-PAGE [11, 13, 14]. Reference proteome maps of many bacteria including *Y. pestis* have been created, and this work is leading to an understanding of the virulence mechanisms and the regulatory networks used by pathogenic bacteria [15]. However, for fish pathogens, in-depth proteomic analysis is not yet well established.

In our previous study, we compared two culture conditions of *Y. ruckeri* strains and focused only on proteins expressed in response to iron-limited culture conditions [16]. In this study, we identified, quantified and analyzed the global proteomic profiles of *Y. ruckeri* strains grown under standard culture conditions using a shotgun proteomic approach. Furthermore, we predicted virulence proteins and antibiotic resistance ontology in the proteome of *Y. ruckeri.*

## Materials and methods

### Bacterial strains

Two biotype 1 (SP-05 and CSF007-82) and two biotype 2 (7959-11 and YRNC-10) *Y. ruckeri* strains were used in the present study. These four strains were isolated from rainbow trout (*Oncorhynchus mykiss*) and all are serotype 01. Strains SP-05 and 7959-11 originated from Austria and the other two strains, CSF007-82 and YRNC-10, originated from the USA. Virulence for rainbow trout was determined previously using an experimental challenge model. Strains CSF007-82, 7959-11 and YRNC-10 were virulent [17, 18] and strain SP-05 was not virulent (Authors unpublished data). The antimicrobial susceptibility of strains was tested using routine clinical laboratory susceptibility methods employing antimicrobial discs [(enrofloxacin (5 µg), florfenicol (30 µg), tetracycline (30 µg), amoxicillin (10 µg), oxolinic acid (2 µg), trimethoprim–sulfamethoxazole (25 µg), flumequine (30 µg) and doxycycline (30 µg)].

### Culture conditions

The culture conditions and growth yield of *Y. ruckeri* strains have been previously described [16]. Briefly, a single colony of each strain was used to inoculate duplicate 5 mL tryptic soy broth cultures. Duplicate starter cultures of each strain (OD_600_ 0.10) were then used to inoculate 25 mL tryptic soy broth cultures and grown overnight at 22 °C until the late log phase. The yield of CSF007-82, 7959-11 and YRNC-10 strains (OD_600_ 1.62) were similar to each other but the yield of SP-05 strain was slightly lower (OD_600_ 1.32) compared to the other three strains [16]. Cells were harvested and washed three times with sterile phosphate buffered saline containing bacterial protease inhibitor cocktail.

### Protein extraction and digestion

The protein extraction procedures used have been previously described [16]. Briefly, bacterial cells were resuspended in denaturing lysis buffer (7 M urea, 2 M thiourea, 4% 3-[(3-cholamidopropyl)dimethyl-ammonio]-1-propane sulfonate and 1% dithiothreitol) containing bacterial protease inhibitor cocktail. Cells were then sonicated on ice and cellular debris removed by centrifugation. Protein digestion was performed using the standard two-step in-solution digestion protocol for Trypsin/LysC mix according to the user manual (Promega) and digested samples were acidified.

### Nano LC–MS/MS analysis

Tryptic peptides were separated by a nano liquid chromatography system (Dionex Ultimate 3000 RSLC) and analyzed with a high-resolution hybrid triple quadrupole time of flight mass spectrometer (TripleTOF 5600+, Sciex) coupled via a nano-ESI interface. Preconcentration and desalting of samples were accomplished with a 5 mm Acclaim PepMap µ-Precolumn (Dionex). Details of the LC–MS/MS procedure were described previously [16]. Briefly, 370 ng of digested protein were used per injection and peptide separation was performed on a 25 cm Acclaim PepMap C18 column with a flow rate of 300 nL/min. The gradient started with 4% mobile phase B (80% acetonitrile with 0.1% formic acid) and increased to 35% B over 120 min. MS1 survey scans were collected in the range of 400–1500 mass-to-charge ratio (m/z). The 25 most intense precursors with charge state 2–4, which exceeded 100 counts per second, were selected for fragmentation for 250 ms. MS2 product ion scans were collected in the range of 100–1800 m/z for 110 ms. Precursor ions were dynamically excluded from reselection for 12 s.

For quantitative measurements, data independent sequential window acquisition of all theoretical spectra (SWATH) technology based on MS2 quantification was used [19, 20]. Peptides from biological and technical replicates were fragmented in 35 fixed fragmentation windows of 20 Dalton (Da) in the range of 400–1100 Da with an accumulation time of 50 ms in TOF MS mode and 80 ms in product ion mode. The nano-HPLC system was operated by Chromeleon 6.8 (Dionex) and the MS by Analyst Software 1.6 (Sciex).

### Data analysis

Database searches of raw files of data dependent acquisition were carried out with Protein Pilot Software version 5.0 (Sciex). UniProt database (Released 10_2016) was restricted to *Y. ruckeri*. Mass tolerance in MS mode was set with 0.05 and 0.1 Da in MS/MS mode for the rapid recalibration search as well as 0.0011 Da in MS and 0.01 Da in MS/MS mode for the final search. The following sample parameters were applied: trypsin digestion, cysteine alkylation set to iodoacetamide and the search effort set was to rapid identification. False discovery rate analysis was performed using the integrated tools in ProteinPilot. The global false discovery rate (FDR) was set to < 1% on the protein level, peptide level as well as spectra level. Information dependent data acquisition identification results were used to create the SWATH ion library with the MS/MS (ALL) with SWATH Acquisition MicroApp 2.0 in PeakView 2.2 (both Sciex). Peptides were chosen based on a FDR rate < 1%, excluding shared and modified peptides. Up to six peptides per protein and up to 6 transitions per peptide were used. MarkerView 1.2.1 (Sciex) was used for calculation of peak areas of SWATH samples after retention time alignment and normalization using total area sums. The resulting protein lists were then used for visualization of data after principal component analysis (PCA) in form of loading plots and score plots to get a first impression of the overall data structure and to assess variability between technical and biological replicates.

Differentially expressed proteins were determined by statistical analysis in R programming language [21]. Raw peak areas after normalization to total area sums were log_2_-transformed to approach a normal distribution. On a logarithmic scale, technical replicates were aggregated by arithmetic mean before application of statistical tests. This procedure is equivalent to the application of a hierarchical model in the subsequent ANOVA, as the same number of technical replicates was measured per biological replicate. Differential expression of proteins in each strain was assessed using one-way ANOVA for each protein. To adjust for multiple testing, the method of Benjamini and Hochberg [22] was used to control the FDR. Differences were considered significant if adjusted *p*-values were smaller than the significance level of *α* = 0.001. For those proteins, Tukey’s honest significant difference method was applied as post hoc test to assess the significance of the pairwise comparisons. Protein expression was considered differential if the adjusted *p-*value was below *α* and the absolute fold change was at least three (fold change < −3 or > +3).

### GO annotation and prediction of virulent proteins

Venn diagrams were used to show the differences between protein lists originating from different strains [23]. Gene ontology annotation of all identified proteins was classified using the software tool for researching annotations of proteins [24]. Subcellular localization of proteins was predicted by PSORTb version 3.0 [25]. Virulence proteins were predicted by a method based on bi-layer cascade Support Vector Machine using VirulentPred [26].

### Antibiotic resistance ontology and their validation

Antibiotic resistance ontology was identified using a comprehensive antibiotic resistance database [27]. The antibiotic resistance phenotypes predicted by in silico analysis were validated using the disc diffusion technique and minimal inhibitory concentration (MIC) determination [28, 29]. The antimicrobial commercial Oxoid discs (µg disc/mL, Thermo Scientific): gentamicin (10 µg), polymyxin B (300 UI), erythromycin (15 µg), rifampin (5 µg), novobiocin (5 µg) and mupirocin (5 µg) were applied to inoculated Mueller–Hinton agar (Thermo Scientific) in triplicate. In parallel, MIC ranges for the same antibiotics were determined using microtiter plates and solutions of antibiotics prepared from powders of known potencies (Sigma-Aldrich). All plates were incubated for 48 h at 22 °C. The diameter of the inhibition halo of antimicrobial discs and lowest concentration of antibiotic that inhibited visible growth of bacteria were defined and categorized as susceptible or resistant (Additional file 1) as previously using standard methods [28, 29].

## Results

### Protein identification

**Figure 1:**
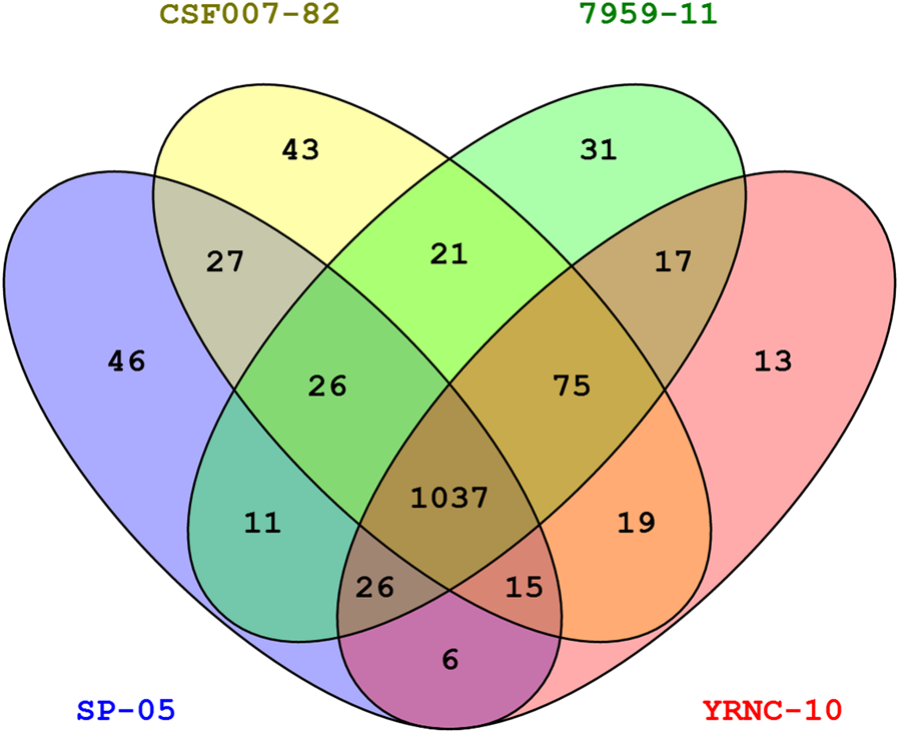
**Venn diagram showing the number of proteins identified in** ***Yersinia ruckeri*** **strains.** The number of unique or shared proteins in each strain is indicated in each set or subset.

A total of 1395 proteins in the whole cell of *Y. ruckeri* were identified (Additional file 2). The number of proteins identified in each strain was 1193 for SP-05, 1263 for CSF007-82, 1244 for 7959-11 and 1208 for YRNC-10. The list of identified proteins in each strain is given in Additional file 3. Forty-six proteins in SP-05, 43 proteins in CSF007-82, 31 proteins in 7959-11 and 13 proteins in YRNC-10 were uniquely identified (Figure 1). PCA score plots of all strains suggested that strain SP-05 differs from the other three strains (CSF007-82, 7959-11 and YRNC-10) but the latter three strains showed minor proteomic differences (Additional file 4). The list of uniquely identified proteins in each strain is given in Additional file 5.

### Protein quantification

Sophisticated statistical evaluation revealed a total number of 36 differentially expressed proteins within the four analyzed *Y. ruckeri* strains. Of these, 16 were upregulated (SP-05 strain versus the other strains) (Table 1) and 20 were downregulated (Additional file 6). As can be seen in Table 1, upregulated proteins were related to iron ion homeostasis, regulation of transcription, transporter activity and metabolic processes. Similarly, downregulated proteins were related to flagellar motility, phosphotransferase system, glycolysis and metabolic processes. We observed upregulation of two proteins: phosphoenolpyruvate (> 25.1-fold) and asparagines synthase (4.3-fold) in biotype 2 strains [biotype 1 strain (CSF007-82) versus biotype 2 strains (7959-11 and YRNC-10)] but saw no significant expression differences between biotype 2 strains (7959-11 versus YRNC-10).

**Table 1:** **Fold changes of differentially expressed proteins of** ***Yersinia ruckeri*** **strains compared to each other**

UniProt accession number	Protein	Function	SP-05 vs CSF007-82	SP-05 vs 7959-11	SP-05 vs YRNC-10	CSF007-82 vs 7959-11	CSF007-82 vs YRNC-10	7959-11 vs YRNC-10
A0A085U6V7_YERRU	Bacterioferritin	Ferric iron binding	6.8*	5.7*	6.5*	−1.2	−1.0	1.1
A0A085U4B6_YERRU	DNA protection during starvation protein	Iron ion homeostasis	3.2*	3.8*	1.9	1.2	−1.7	−2.1
A0A085U5L5_YERRU	Anti-sigma factor antagonist	Regulation of transcription	3.6*	3.7*	3.8*	1.0	1.1	1.0
A0A085U5L7_YERRU	Anti-sigma regulatory factor	Serine/threonine kinase activity	3.9*	3.9*	4.3*	−*1.0*	1.1	1.1
A0A085UBQ1_YERRU	Arginine deiminase	Arginine catabolic process	5.7*	5.2*	5.8*	−1.1	1.0	1.1
A0A085U605_YERRU	Amino acid transporter	Transporter activity	4.3*	4.2*	4.0*	−1.0	−1.1	−1.0
A0A085U8U0_YERRU	Phosphate-binding protein PstS	Phosphate ion transmembrane transport	3.0	3.1*	3.4*	1.0	1.1	1.1
A0A0A5FQB4_YERRU	Superoxide dismutase Cu–Zn	Superoxide dismutase activity	2.8	3.2*	3.4*	1.2	1.2	1.1
A0A0A5FMC5_YERRU	Arginine decarboxylase, catabolic	Amino acid metabolic process	7.9*	6.5*	6.2*	−1.2	−1.3	−1.0
A0A085UBP8_YERRU	Glutamate decarboxylase	Glutamate metabolic process	10.4*	8.5*	6.8*	−1.2	−1.5	−1.3
A0A0A8VE52_YERRU	Glutaminase	Glutamine metabolic process	6.7*	6.5*	8.2*	−1.0	1.2	1.3
A0A085U745_YERRU	Glucose-1-phosphate adenylyltransferase	Glycogen biosynthetic process	5.3*	5.0*	3.8*	−1.1	−1.4	−1.3
A0A085UBM7_YERRU	3-Oxoacyl-ACP reductase	Oxidoreductase	6.2*	6.1*	6.8*	−1.0	1.1	1.1
A0A085U7G0_YERRU	Uncharacterized protein	Unknown	9.1*	7.9*	9.5*	−1.2	1.0	1.2
A0A085UBQ0_YERRU	Uncharacterized protein	Unknown	5.0*	4.9*	4.3*	−1.0	−1.2	−1.2
A0A085U732_YERRU	Putative exported protein	Unknown	2.9	2.9	3.4*	1.0	1.2	1.2

ANOVA was performed for UniProt database searches.

* Denotes statistically significant difference according to Tukey’s honest significant difference post hoc test with FDR-adjusted *p* < 0.001 and fold change < −3 or > +3.

### GO annotation and subcellular localization of proteins

**Figure 2:**
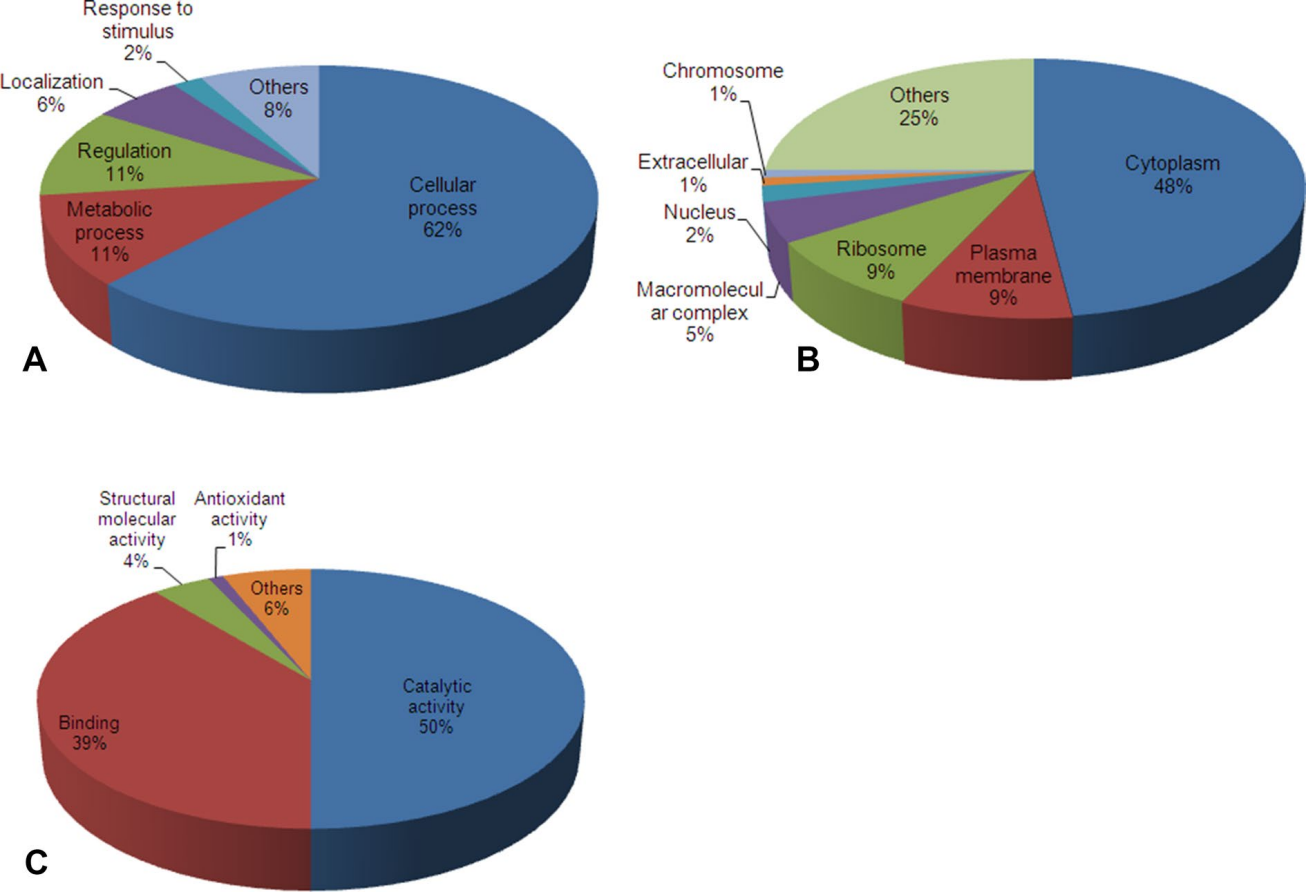
**Proteomic profiles of identified proteins of** ***Yersinia ruckeri***. Proteins were classified by gene ontology terms for biological processes, cellular components and molecular functions using software tool for researching annotations of proteins (**A**) biological process, (**B**) cellular component, and (**C**) molecular function.

**Figure 3:**
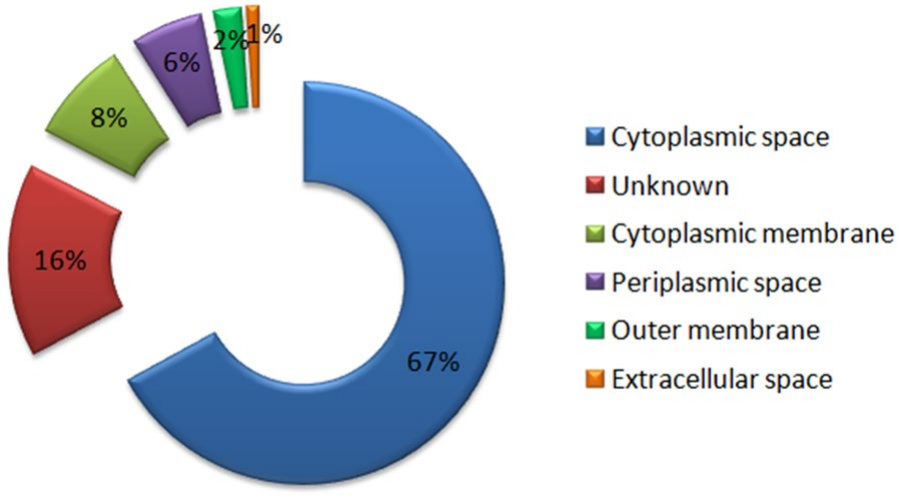
**Subcellular locations of** ***Yersinia ruckeri*** **proteins.** Cellular location of proteins was predicted by PSORTb version 3.0 and particular location of proteins was shown in percentage. Unknown location includes proteins with multiple localization sites or unknown location.

The identified proteins were associated with cellular process, metabolic process, regulation, localization and response to stimulus (Figure 2A). Proteins were localized in the cytoplasm, plasma membrane, ribosome, macromolecular complex, nucleus, chromosome and others (Figure 2B). Proteins involved in catalytic activity and binding were the most abundant among those identified proteins, 51 and 39%, respectively (Figure 2C). The identified proteins were predicted in the cytoplasmic space (67%), unknown (16%), cytoplasmic membrane (8%), periplasmic space (6%), outer membrane (2%) and extracellular space (1%) (Figure 3). The unknown group included proteins with multiple subcellular and unknown localizations.

### Virulence proteins and antibiotic resistance ontology

Several predicted virulence proteins were identified: HtrA protease, protein TolB, peptidyl-prolyl cis–trans isomerase, UvrY response regulator, chaperone protein fimC, lipoprotein NlpD, putative exported protein, MltA-interacting protein, superoxide dismutase Cu–Zn, PhoP, LuxR and AsnC family transcriptional regulators (Table 2 and Additional file 7).

**Table 2:** **Lists of important virulence proteins of** ***Yersinia ruckeri***

Protein	Function	Cascade of SVMs and PSI-BLAST, score
Gene expression modulator/haemolysin expression modulating protein	Haemolysin expression	1.0520
HtrA protease	Serine-type endopeptidase activity	0.5097
Outer membrane stress sensor protease DegQ serine protease	Serine-type endopeptidase activity	1.0012
Anti-sigma regulatory factor	Protein serine/threonine kinase activity	0.9363
Beta-barrel assembly-enhancing protease	Chaperone and a metalloprotease	1.0339
BarA-associated response regulator UvrY	Regulation of transcription	0.9837
Peptidyl-prolyl cis–trans isomerase	Protein folding	0.9128
PhoP family transcriptional regulator	Regulation of transcription	1.0898
LuxR family transcriptional regulator	Regulation of transcription	1.0031
AsnC family transcriptional regulator	Regulation of transcription	0.7053
RNA-binding protein Hfq	Regulation of transcription	1.0260
Anti-sigma factor antagonist	Regulation of transcription	0.9804
Attachment invasion locus protein	Invasion	1.0123
Invasin	Cell adhesion	1.0130
Superoxide dismutase Cu–Zn	Superoxide dismutase activity	1.0003
Molybdenum ABC transporter periplasmic molybdenum-binding protein ModA	Transporter activity	0.8399
DcrB protein	Required for phage C1 adsorption	1.0009
Methyl-accepting chemotaxis protein I	Chemotaxis	0.9279
Methyl-accepting chemotaxis protein III	Chemotaxis	0.9922

Proteins were predicted by a method based on bi-layer cascade support vector machine using VirulentPred.

We also predicted antibiotic resistance ontology in 12 antibiotic classes (Table 3) in the proteome of *Y. ruckeri*, which contains 14 proteins such as bacterial regulatory protein (cyclic AMP receptor protein), membrane fusion protein of the resistance-nodulation-division (RND) family multidrug efflux pump, bifunctional polymyxin resistance protein ArnA and RND efflux system inner membrane transporter CmeB.

**Table 3:** **Details of antibiotic resistance ontology of** ***Yersinia ruckeri***

Protein	Antibiotic resistance ontology	Bit score
Bacterial regulatory, crp family protein (cyclic AMP receptor protein)	Fluoroquinolone (enrofloxacin), beta-lactam (amoxicillin), Macrolide (erythromycin)	431.409
Cys regulon transcriptional activator CysB	Aminocoumarin (novobiocin)	620.928
Copper-sensing two-component system response regulator CpxR	Aminoglycoside (gentamicin), aminocoumarin (novobiocin)	389.808
Alanine tRNA ligase	Aminocoumarin	1487.63
Transcription repair-coupling factor	Fluoroquinolone	1938.7
Dihydropteroate synthase	Sulfonamide	440.654
Membrane fusion protein of RND family multidrug efflux pump	Fluoroquinolone, beta-lactam, Macrolide, Rifampin, Chloramphenicol, Tetracycline, Aminocoumarin	548.125
Beta-lactamase	beta-lactam (amoxicillin)	608.601
Outer membrane channel protein	Fluoroquinolone, beta-lactam, Macrolide, Rifampin, Chloramphenicol, Tetracycline, Aminocoumarin	689.878
Elongation factor Tu	Elfamycin	583.178
Bifunctional polymyxin resistance protein ArnA	Polymyxin B	951.814
DNA gyrase subunit A	Fluoroquinolone	751.895
RND efflux system inner membrane transporter CmeB	Fluoroquinolone, tetracycline	751.125
Isoleucine-tRNA ligase	Mupirocin	219.55

Antibiotic resistance ontology was predicted in the proteome of *Y. ruckeri* using a comprehensive antibiotic resistance database.

### Antimicrobial susceptibility test

Strains of *Y. ruckeri* were susceptible to enrofloxacin, florfenicol, tetracycline, amoxicillin, oxolinic acid, trimethoprim–sulfamethoxazole, flumequine and doxycycline (data not shown). Additional file 1 shows the diameter of the inhibition zone and MIC of antimicrobial agents used for validation of antibiotic resistance ontology. Novobiocin and mupirocin discs displayed no inhibition zone against *Y. ruckeri* strains, while strains showed intermediate susceptibility to gentamicin and polymyxin B. *Y. ruckeri* strains were resistant to erythromycin (MIC = 1024 μg/mL), rifampin (MIC = 32 μg/mL), novobiocin (MIC = 16–32 μg/mL) and mupirocin (MIC = 32–64 μg/mL). Three antibiotic resistance ontologies: novobiocin, mupirocin and erythromycin were fully consistent with the proteomic data such as cys regulon transcriptional activator CysB, alanine tRNA ligae and isoleucine-tRNA ligase.

## Discussion

Here we identify global proteomic reference profiles of *Y. ruckeri* strains (PXD005439) grown under standard culture conditions. These global proteomic profiles help us to understand the physiology, protein biology, virulence factors, host-interactions, localization and antibiotic resistance of *Y. ruckeri*. The total number of proteins identified was 1395 in *Y. ruckeri* (Additional file 2). These included proteases, chaperones, cell division proteins, outer membrane proteins, chromosome partitioning proteins and transporters. Proteins have been classified into different functional categories such as biological process and molecular function (Figure 2) and this information will be useful for further studies in the direction of extracellular (flagellin and flagellar hook-associated protein), interaction with cells (invasin and manganese ABC transporter, periplasmic-binding protein SitA), antioxidant (thioredoxin reductase and glutathione amide-dependent peroxidase) and molecular transducer (methyl-accepting chemotaxis protein) activity. The identification of predicted virulence proteins (Table 2; Additional file 7) and antibiotic resistance ontology (Table 3) contributes to our understanding of this pathogen and will aid in the rational design of novel treatment strategies for ERM disease.

Biotype 2 strains showed minor proteomic differences among each other (Additional file 4). However, the Austrian biotype 1 strain (SP-05) showed major proteomic differences when compared to the USA biotype 1 strain (CSF007-82) and biotype 2 strains (7959-11 and YRNC-10). These major differences may be due to the slightly lower yield and growth rate of SP-05 strain compared to the other three strains (CSF007-82, 7959-11 and YRNC-10) or its avirulent nature toward the fish.

Sixteen upregulated proteins were identified in virulent *Y. ruckeri* strains using a sophisticated statistical analysis (avirulent SP-05 strain versus virulent strains). We found strong upregulation of bacterioferritin (5.7- to 6.8-fold) and DNA protection during starvation protein (3.2- to 3.8-fold) in *Y. ruckeri* strains. However, iron dependent proteins (bacterioferritin and iron-sulfur cluster assembly scaffold protein IscU) were downregulated (−3-fold) in *Y. ruckeri* strains in response to iron-limited culture conditions [16]. The phosphate-binding protein PstS is a high affinity phosphate binding protein of the Pst transport system and has been shown to be involved in pathogenesis, invasion and biofilm formation of many bacteria [30]. Superoxide dismutase Cu–Zn is an important for oxidative stress and has been shown to contribute to the pathogenicity of many bacteria [31]. Arginine deiminase protects bacterial cells against the damaging effects of acidic environments and enhances the ability of cells to survive in acidic extracellular conditions [32]. We observed strong upregulation of phosphate-binding protein PstS (> 3-fold), superoxide dismutase Cu–Zn (3.3- to 3.4-fold) and arginine deiminase (5.2- to 5.8-fold) in *Y. ruckeri* strains. Based on the results of the present study, it appears that upregulated proteins (avirulent strain versus virulent strains) such as PstS, SOD-Cu–Zn and arginine deiminase may be involved in the establishment of disease inside the host and the survival of *Y. ruckeri* during the infection process.

Several proteases such as HtrA, Lon, carboxy-terminal, signal peptidase I, La Type II, HslUV, pyrrolidone–carboxylate, FtsH, protease III, Clp, protease 4, putative protease, peptidase B, T and M37 were identified. These proteases were serine, threonine, cysteine, metalloproteinase and ATP-dependent type proteases, and belonged to the C15, M16, M17, M20B, M23, S16, S26, S41A, S49, U32, AAA ATPase and Clp families including PDZ domains. Proteases play critical roles in the invasion of host tissues, contribute to virulence and damage host tissue during infection [33]. The Yrp1 protease of *Y. ruckeri* has been implicated in the hydrolysis of different matrix and muscle proteins of fish and vaccination with Yrp1 elicits a strong protection against the development of enteric redmouth disease [6]. Additionally, the Clp and Lon pro-teases have been shown to have a role in the regulation of the type III secretion systems (T3SS) in various bacterial pathogens. The T3SS forms a needle-like structure in several Gram negative bacteria that allows direct transfer of bacterial virulence factors into the cytoplasm of host cells. The T3SS has been linked to flagellum biosynthesis [34]. We also identified flagellar biosynthesis proteins (FliC, FliG, FliH and FliN), flagellar hook proteins (FlgD, FlgE and FlgK), flagellar brake protein YcgR, flagellar motor protein MotB and pilus assembly protein PilW in *Y. ruckeri*. FliC and FliH flagellar proteins have been linked with pathogenesis in the fish pathogen, *Edwardsiella tarda* [35]. Additionally, the *Y. ruckeri* flagellin protein has been shown to elicit a robust innate immune response and protect fish against biotype 1 and biotype 2 *Y. ruckeri* strains [36]. More research on the role of proteases and T3SS in *Y. ruckeri* virulence is needed to more fully understand the pathogenicity of *Y. ruckeri*.

We also identified other important virulence proteins such as the UvrY response regulator, peptidyl-prolyl cis–trans isomerase (PPIases), TolB, PhoP and LuxR family transcriptional regulators. UvrY is a response regulator of the BarA-UvrY two-component system and has been shown to be involved in the pathogenesis of *Y. ruckeri*, probably through its regulation of both the invasion of epithelial cells and protection against oxidative stress induced by immune cells [37]. PPIases are FKBP domain-containing ubiquitous folding proteins and have been reported as virulence factors in several bacterial pathogens [38]. Upregulation of FKBP-type peptidyl-prolyl cis–trans isomerases has been observed in iron-starved biotype 2 *Y. ruckeri* strains [16], which may be involved in virulence of *Y. ruckeri*. PhoP is part of a two component system and is important for bacterial survival and replication in macrophages [39]. TolB is the periplasmic component of the Tol–Pal system and is important for antibiotic resistance and pathogenicity in Gram negative pathogens and has been suggested as a suitable candidate for the development of novel drugs against *Pseudomonas aeruginosa* [40]. The LuxR transcriptional regulator is a key player in quorum sensing and affects survival, virulence, antibiotic biosynthesis and biofilm formation of bacteria [41].

A number of chaperone proteins (CbpA, ClpB, DnaK, DnaJ, fimC, HscA, HscB, HtpG, skp, SurA, ProQ), an acid stress chaperone HdeB, universal stress protein E, cold shock (CspC and CspE) and a phage shock protein were identified in *Y. ruckeri*. Bacterial pathogens produce a number of chaperone proteins for survival during changing environments and stress conditions [42]. Some chaperone proteins have also been implicated in bacterial virulence [43]. DnaK chaperone protein plays a role in protein folding and interacts with ClpB in reactivating proteins which have become aggregated after heat shock [44]. The DnaK/DnaJ chaperone machinery and ClpB have been shown to be involved in the invasion of epithelial cells and survival within macrophages of the host, leading to systemic infection of *Salmonella enterica* and *Francisella tularensis* in mice [43, 45]. Upregulation of ClpB, HtpG and universal stress protein A have been observed in *Flavobacterium psychrophilum* during in vivo growth in fish and were suggested to play an important role in the pathogenesis of *F. psychrophilum* [46]. Based on these data, we suggest that some chaperone proteins may be important for in vivo survival and pathogenesis of *Y. ruckeri*.

A number of cell division proteins (BolA, DedD, DamX, FtsA, FtsE, FtsH, FtsP, FtsZ, ZapA, ZapB and ZapD), chromosome partitioning proteins (ParA, ParB, MukB and MukE) and biosynthesis proteins (iscR, MraZ, basR/pmrA, IF-1, IF-3, S2-S21, L1-L6, RsmA-RsmC and RsmG-RsmI) were identified. The FtsZ and ParA proteins have been identified as potential drug targets against clinically important bacterial pathogens [47]. Protein synthesis (transcriptional and translational) proteins have been targeted for inhibition of bacterial pathogens [48]. However, cell division and chromosome partitioning proteins may act as new drug targets for *Y. ruckeri*. Additionally, we predicted 12 antibiotic resistance classes (Table 3) in the *Y. ruckeri* proteome, particularly for cys regulon transcriptional activator CysB, bifunctional polymyxin resistance protein ArnA, copper-sensing two-component system response regulator CpxR and isoleucine-tRNA ligase. We observed intermediate susceptibility of aminoglycoside (gentamicin, MIC = 4–8 μg/mL) and polymyxin B (MIC = 4 μg/mL) antibiotics against *Y. ruckeri* strains. Similar results were previously reported in French *Y. ruckeri* isolates with aminoglycoside (gentamicin) [49] and greatest variation (MIC = 2–512 μg/mL) in antibiotic sensitivity of polymyxin B was reported among *Y. ruckeri* strains [50]. These higher MIC values suggest that *Y. ruckeri* strains may harbor acquired or intrinsic resistance mechanisms to aminoglycosides and polymyxin B. Additionally, our *Y. ruckeri* strains were highly resistant to erythromycin (MIC = 1024 μg/mL) and rifampin (MIC = 32 μg/mL), consistent with observations by Calvez et al. [49] and Stock et al. [51], who found *Y. ruckeri* strains to be resistant to erythromycin (MIC = 32–64 μg/mL) and rifampin (MIC = 8–16 μg/mL). Erythromycin and novobiocin discs did not show inhibition zone against the Chinese *Y. ruckeri* strain H01 [52]. Similarly, we did not observe any inhibition zone of novobiocin and mupirocin discs against the *Y. ruckeri* strains examined. Inherent resistance to erythromycin and rifampin has been described for the other *Yersinia* species (*Y. enterocolitica, Y. mollaretii* and *Y. aldovae*) [51]. Our results support these findings and suggest that *Y. ruckeri* strains might also be resistant to novobiocin and mupirocin. Moreover, two efflux pumps of the RND family were identified. This family is widespread among Gram negative bacteria and, in Enterobacteriaceae such as *E. coli*, contributes to the intrinsic resistance against several antibiotics, including macrolide and novobiocin [53]. This is consistent with our present results that found *Y. ruckeri* to be resistant to both antibiotics. Finally, it is important to note that the antimicrobial agents used to validate the results of our antibiotic resistance ontology are generally not approved for use in aquaculture. *Y. ruckeri* strains are susceptible to commonly applied antimicrobial agents such as florfenicol and oxytetracycline to treat fish diseases [49].

The outer membrane proteins (OmpA, OmpC, OmpF and OmpW), outer membrane assembly factors (BamA, BamB, BamC, BamD and BamE), outer membrane lipoproteins (Blc, pcp, RcsF, LolB, LolD, Omp16, RcsF and YfeY), lipoproteins (NlpD, NlpE and NlpI) and lipopolysaccharide biosynthesis proteins (LptA, LptD and LptE) were identified. These proteins play an important role in pathogen-host interactions and pathogenicity [54]. Additionally, OMPs help in resisting host defense mechanisms and have been shown to confer protection in fish [54, 55]. The outer membrane assembly factor YeaT and OmpC have been shown to induce a strong immune response and protect *Labeo rohita* and *Japanese flounder* against *Edwardsiella tarda* infection [56, 57].

In conclusion, our study provides the first global proteomic profiles of *Y. ruckeri* and this work will provide a better understanding of the physiology, proteomic biology, proteomic changes, virulence mechanisms and localization of *Y. ruckeri* proteins. The most commonly expressed proteins such as SOD-Cu–Zn and PstS might be useful to develop a single vaccination protocol or single drug therapy for both biotype 1 and biotype 2 strains. Additionally, proteins associated with virulence and antigenicity such as Clp and Lon pro-teases, TolB, PPIases, PhoP and LuxR family transcriptional regulators may be used for the construction of novel vaccines for yersiniosis in fish. The comprehensive data set generated in this study will serve as a reference proteome for future studies such as protein–protein interaction and network analysis.

### Data deposition

Shotgun proteomics data have been deposited in the ProteomeXchange Consortium (http://proteomecentral.proteomexchange.org) via the PRIDE partner repository [58] with the dataset identifier PXD005439.

## Additional files

**Additional file 1.** **Antimicrobial susceptibility of** ***Yersinia ruckeri*** **strains.** Antibiotic susceptibility was determined using the disc diffusion technique on Mueller–Hinton agar and minimal inhibitory concentration was determined with the same antibiotics using micro dilution on microtiter plates. The diameter of the inhibition halo and lowest concentration of antibiotic that inhibited visible growth of bacteria was defined after incubation 48 h at 22 °C. Novobiocin and mupirocin discs displaced no inhibition zone against *Y. ruckeri* strains. Note: I = intermediate and R = resistant.

**Additional file 2.** **Details of total identified proteins of** ***Yersinia ruckeri***. Number of proteins was identified at false discovery rate 1% with more than one peptide.

**Additional file 3.** **Details of identified proteins of** ***Yersinia ruckeri*** **strains.** Number of proteins was identified at false discovery rate 1% with more than one peptide.

**Additional file 4.** **Principal component analysis of** ***Yersinia ruckeri*** **strains.** The score plots show that strain SP-05 differs from the three strains (CSF007-82, 7959-11 and YRNC-10) but the latter three strains showed minor proteomic differences.

**Additional file 5.** **Lists of uniquely identified proteins in each strain of** ***Yersinia ruckeri***. Forty-six proteins in SP-05, 43 proteins in CSF007-82, 31 proteins in 7959-11 and 13 proteins in YRNC-10 were uniquely identified.

**Additional file 6.** **Fold changes of differentially down regulated proteins of** ***Yersinia ruckeri*** **strains compared to each other.** ANOVA was performed for UniProt database searches. * Denotes statistically significant difference according to Tukey’s honest significant difference post hoc test with false discovery rate-adjusted *p*-value < 0.001 and fold change < −3 or > +3.

**Additional file 7.** **Lists of virulence proteins of** ***Yersinia ruckeri.*** Proteins were predicted by a method based on bi-layer cascade support vector machine using VirulentPred.

## Abbreviations

ERM: enteric redmouth disease
LC–MS: liquid chromatography–mass spectrometry
TOF: triple quadrupole time of flight
FDR: false discovery rate
SWATH: sequential window acquisition of all theoretical spectra
IDA: information dependent data acquisition
PCA: principal component analysis
