# Supplementary material for: Global proteomic profiling of Yersinia ruckeri strains
Source: Vet Res. 2017 Sep 20;48:55. doi: 10.1186/s13567-017-0460-3 (PMC5607619; doi:10.1186/s13567-017-0460-3)
Supplement: Supplementary file 1 — Additional file 1. Antimicrobial susceptibility of Yersinia ruckeri strains. Antibiotic susceptibility was determined using the disc diffusion technique on Mueller–Hinton agar and minimal inhibitory concentration was determined with the same antibiotics using micro dilution on microtiter plates. The diameter of the inhibition halo and lowest concentration of antibiotic that inhibited visible growth of bacteria was defined after incubation 48 h at 22 °C. Novobiocin and mupirocin discs displaced no inhibition zone against Y. ruckeri strains. Note: I = intermediate and R = resistant. [file 13567_2017_460_MOESM1_ESM.doc]

**Additional file 1: Antimicrobial susceptibility** **of *Yersinia ruckeri* strains.** Antibiotic susceptibility was determined using the disc diffusion technique on Mueller-Hinton agar and minimal inhibitory concentration was determined with the same antibiotics using micro dilution on microtiter plates. The diameter of the inhibition halo and lowest concentration of antibiotic that inhibited visible growth of bacteria was defined after incubation 48 hours at 22 °C. Novobiocin and mupirocin discs displaced no inhibition zone against *Y. ruckeri* strains.

Note: I = intermediate and R = resistant.

| **Antimicrobial agent** | **Disk content**  **(µg disc/ml)** | **Zone diameter (mm)** | | ***Y. ruckeri* inhibition zone (mm)** | **MIC break point (µg/ml)** | | ***Y. ruckeri* MIC**  **(µg/ml)** | **Final result** |
| --- | --- | --- | --- | --- | --- | --- | --- | --- |
|  |  | **Susceptible** | **Resistant** |  | **Susceptible** | **Resistant** |  |  |
| Gentamicin | 10 μg | ≥21 | ≤12 | 14−15 | ≤2 | ≥8 | 4−8 | I |
| Polymyxin B | 300 UI | ≥15 | ≤11 | 11−12 | ≤1 | ≥4 | 4 | I |
| Erythromycin | 15 µg | ≥23 | ≤13 | 12−13 | ≤0.5 | ≥8 | 1024 | R |
| Rifampin | 5 µg | ≥20 | ≤16 | 11−12 | ≤1 | ≥4 | 32 | R |
| Novobiocin | 5 µg | ≥16 | ≤12 | 0 | ≤2 | ≥8 | 16−32 | R |
| Mupirocin | 5 µg | ≥18 | ≤13 | 0 | ≤2 | ≥8 | 32−64 | R |
