# Supplementary figures and images for: Global proteomic profiling of Yersinia ruckeri strains
Source: Vet Res. 2017 Sep 20;48:55. doi: 10.1186/s13567-017-0460-3 (PMC5607619; doi:10.1186/s13567-017-0460-3)

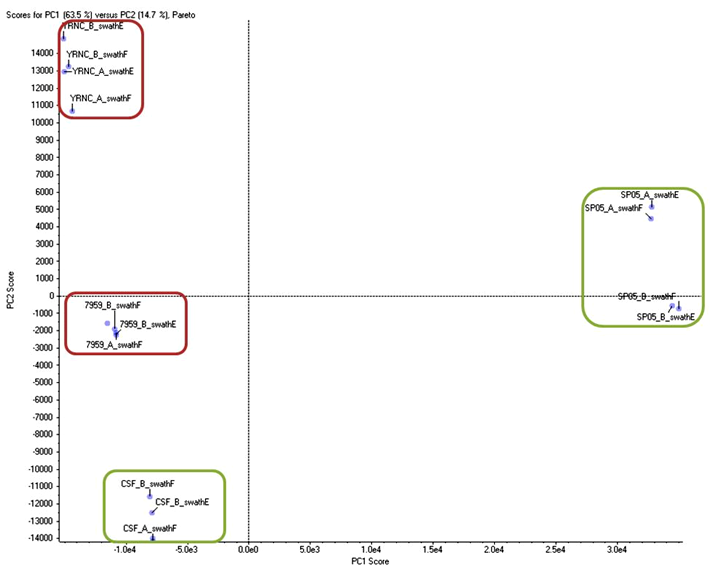

Supplement: Supplementary file 4 — Additional file 4. Principal component analysis of Yersinia ruckeri strains. The score plots show that strain SP-05 differs from the three strains (CSF007-82, 7959-11 and YRNC-10) but the latter three strains showed minor proteomic differences. [file 13567_2017_460_MOESM4_ESM.tif]
