# Supplementary material for: Global proteomic profiling of Yersinia ruckeri strains
Source: Vet Res. 2017 Sep 20;48:55. doi: 10.1186/s13567-017-0460-3 (PMC5607619; doi:10.1186/s13567-017-0460-3)
Supplement: Supplementary file 6 — Additional file 6. Fold changes of differentially down regulated proteins of Yersinia ruckeri strains compared to each other. ANOVA was performed for UniProt database searches. * Denotes statistically significant difference according to Tukey’s honest significant difference post hoc test with false discovery rate-adjusted p-value < 0.001 and fold change < −3 or > +3. [file 13567_2017_460_MOESM6_ESM.doc]

**Additional file 6:** Fold changes of differentially down regulated proteins of *Y. ruckeri* strains compared to each other. ANOVA was performed for UniProt database searches. * denotes statistically significant difference according to Tukey’s honest significant difference post hoc test with false discovery rate-adjusted *p*-value < 0.001 and fold change < − 3 or > +3.

| **UniProt  Accession number** | **Protein** | **Function** | **SP-05**  versus  **CSF007-82** | **SP-05**  versus  **7959-11** | **SP-05**  versus  **YRNC-10** | **CSF007-82**  versus  **7959-11** | **CSF007-82** versus  **YRNC-10** | **7959-11** versus  **YRNC-10** |
| --- | --- | --- | --- | --- | --- | --- | --- | --- |
| A0A085U984_YERRU | Ornithine decarboxylase | Amino acid metabolic process | **-5.5*** | **-5.5*** | **-6.1*** | -1.0 | -1.1 | -1.1 |
| A0A085UAK7_YERRU | NADP-dependent malic enzyme | Metabolic process | **-3.6*** | **-3.8*** | **-3.5*** | -1.1 | 1.0 | 1.1 |
| A0A085U3M7_YERRU | Glycerol kinase | Metabolic process | **-4.7*** | **-6.1*** | **-5.4*** | -1.3 | -1.1 | 1.1 |
| A0A085U9J0_YERRU | Aspartate ammonia-lyase | Metabolic process | **-4.2*** | **-4.1*** | **-5.1*** | 1.0 | -1.2 | -1.2 |
| A0A085U4X4_YERRU | PTS ascorbate transporter subunit IIB | Phosphotransferase system | **-3.9*** | **-4.6*** | **-3.9*** | -1.2 | -1.0 | 1.2 |
| A0A085U489_YERRU | Allophanate hydrolase 2 subunit 1 | Allophanate hydrolase activity | **-10.8*** | **-9.6*** | **-10.0*** | 1.1 | 1.1 | -1.0 |
| A0A085UB64_YERRU | Glycine cleavage system H protein | Glycine cleavage system | **-3.2*** | **-3.2*** | **-3.1*** | -1.0 | 1.0 | 1.0 |
| A0A0A8VEE9_YERRU | Phosphoenolpyruvate carboxykinase | Gluconeogenesis | **-123.2*** | **-4.9*** | **-4.7*** | **25.1*** | **26.2*** | 1.0 |
| A0A085U8W7_YERRU | Flagellin | Structural molecule activity | **-5.1*** | **-128.3*** | **-120.6*** | **-25.3*** | **-23.8*** | 1.1 |
| A0A0A8VGZ8_YERRU | Asparagine synthase | Asparagine synthase activity | **-3.9*** | 1.1 | **-4.3*** | **4.3*** | -1.1 | **-4.8*** |
| A0A085U8Y4_YERRU | Flagellar motor switch protein FliM | Motor activity | **-3.4*** | **-3.9*** | **-6.0*** | -1.1 | -1.7 | -1.5 |
| A0A0A8VDU4_YERRU | Flagellar biosynthesis protein FliC | Structural molecule activity | -3.0 | **-109.8*** | **-78.8*** | **-36.6*** | **-26.3*** | 1.4 |
| A0A085UB63_YERRU | Aminomethyltransferase | Aminomethyltransferase activity | **-3.3*** | **-3.2*** | **-3.9*** | 1.0 | -1.2 | -1.2 |
| A0A085U4W3_YERRU | HTH-type transcriptional regulator pecT | Transcription | **-12.7*** | **-16.8*** | **-13.2*** | -1.3 | -1.0 | 1.3 |
| A0A085U923_YERRU | Chemotaxis protein CheY | Signal transduction system | -2.6 | **-9.3*** | **-9.6*** | **-3.5*** | **-3.6*** | -1.0 |
| A0A085U669_YERRU | PTS mannose transporter subunit IIAB | Phosphotransferase system | **-3.2*** | -2.7 | -2.9 | 1.2 | 1.1 | -1.1 |
| A0A085U5X1_YERRU | LacI family transcriptional regulator | Transcription | -2.4 | **-3.5*** | -3.0 | -1.4 | -1.2 | 1.2 |
| A0A085U2E6_YERRU | Glucokinase | Glycolytic process | -2.4 | -2.8 | **-3.6*** | -1.2 | -1.5 | -1.3 |
| A0A0A8V8I4_YERRU | Methyl-accepting chemotaxis protein I | Chemotaxis | -2.0 | -2.8 | **-3.7*** | -1.4 | -1.8 | -1.3 |
| A0A085U668_YERRU | PTS mannose transporter subunit IIC | Phosphotransferase system | -2.9 | -2.1 | **-4.8*** | 1.4 | -1.7 | -2.3 |
