# Supplementary material for: Global proteomic profiling of Yersinia ruckeri strains
Source: Vet Res. 2017 Sep 20;48:55. doi: 10.1186/s13567-017-0460-3 (PMC5607619; doi:10.1186/s13567-017-0460-3)
Supplement: Supplementary file 7 — Additional file 7. Lists of virulence proteins of Yersinia ruckeri. Proteins were predicted by a method based on bi-layer cascade support vector machine using VirulentPred. [file 13567_2017_460_MOESM7_ESM.doc]

**Additional file 7: Lists of virulence proteins of *Yersinia ruckeri.* Proteins were predicted by a method based on bi-layer cascade Support Vector Machine using VirulentPred.**

| **Accession number** | **Protein** | **Cascade of SVMs and PSI-BLAST, score** |
| --- | --- | --- |
| **Flagella** |  |  |
| A0A085U8W7_YERRU | Flagellin | 1.0066 |
| A0A085U8Z1_YERRU | Flagellar hook-associated protein | 1.0387 |
| A0A0A8VHY4_YERRU | Flagellar hook protein FlgE | 1.0143 |
| A0A085U546_YERRU | Flagellar brake protein YcgR | 0.2046 |
| A0A0A8VJ11_YERRU | Flagellar assembly protein FliH | 1.0338 |
| A0A085U8Y5_YERRU | Flagellar motor switch protein FliN | 0.9161 |
| A0A085U8Z1_YERRU | Flagellar hook-associated protein 1 | 1.3795 |
| A0A085U8W8_YERRU | Flagellar hook-associated protein 2 | 1.0679 |
| A0A085U930_YERRU | Flagellar motor protein MotB | 1.0068 |
| A0A085UAD9_YERRU | Pilus assembly protein PilW | 0.9999 |
| A0A085U8Z8_YERRU | Basal body rod modification protein FlgD | 1.7201 |
| **Outer membrane protein assembly factor** |  |  |
| A0A0A8VG61_YERRU | Outer membrane protein assembly factor BamA | 0.9699 |
| A0A085UAE4_YERRU | Outer membrane protein assembly factor BamB | 0.0590 |
| A0A085UAI7_YERRU | Outer membrane protein assembly factor BamC | 1.1035 |
| A0A0A8VFB3_YERRU | Outer membrane protein assembly factor BamE | 0.9997 |
| **Outer membrane lipoprotein** |  |  |
| A0A085U7U2_YERRU | Outer membrane lipoprotein pcp | 1.0204 |
| A0A085U9H5_YERRU | Outer membrane lipoprotein Blc | 1.0783 |
| A0A085U2W5_YERRU | Outer membrane lipoprotein RcsF | 0.3874 |
| A0A085U9U5_YERRU | Outer membrane lipoprotein carrier protein | 0.9306 |
| A0A085U7W2_YERRU | Major outer membrane lipoprotein | 0.9771 |
| A0A085U893_YERRU | Outer membrane porin protein C | 0.5801 |
| A0A0A8VCQ6_YERRU | Outer membrane lipoprotein LolB | 0.8522 |
| A0A085U2V8_YERRU | Predicted outer membrane lipoprotein YfeY | 0.9428 |
| **Lipoprotein** |  |  |
| A0A085U9S3_YERRU | Lipoprotein | 1.0082 |
| A0A0A8VAG1_YERRU | Hypothetical lipoprotein YajG | 1.0403 |
| A0A085U5P2_YERRU | Rare lipoprotein A | 0.4376 |
| A0A085UAR8_YERRU | Lipopolysaccharide export system protein LptA | 1.0028 |
| A0A085U3A3_YERRU | Lipoprotein NlpD | 0.5011 |
| A0A0A8VBD2_YERRU | LPS-assembly lipoprotein LptE | 0.5913 |
| A0A0A8VIJ3_YERRU | Putative lipoprotein | 1.0685 |
| A0A0A8VLP9_YERRU | Lipopolysaccharide heptosyltransferase | 0.7385 |
| A0A085U2Y2_YERRU | Lipid A disaccharide synthase | 0.8794 |
| A0A0A5FVD2_YERRU | LPS assembly protein LptD | 1.0749 |
| **Channel** |  |  |
| A0A085U3G6_YERRU | Outer membrane channel protein | 1.0720 |
| A0A085U5A0_YERRU | Ion channel protein Tsx | 0.7467 |
| A0A0A8VE91_YERRU | Colicin protein | 0.9146 |
| **Membrane** |  |  |
| A0A085U689_YERRU | Putative membrane protein YebY | 0.9877 |
| A0A085U679_YERRU | Putative membrane protein | 1.0039 |
| A0A0A8VLB3_YERRU | Putative inner membrane protein | 0.5653 |
| A0A0A8V9B5_YERRU | Putative membrane protein yjeI | 1.0170 |
| A0A085UAE3_YERRU | Membrane protein/ Mlr7403 protein | 0.9112 |
| **Chaperone** |  |  |
| A0A085U2Y6_YERRU | Chaperone protein skp | 1.0978 |
| A0A0A8VHL6_YERRU | Chaperone protein fimC | 0.9835 |
| A0A085UAC8_YERRU | Co-chaperone protein HscB | 1.1152 |
| A0A085U761_YERRU | Universal stress protein | 0.9906 |
| A0A0A8VJQ2_YERRU | Acid stress chaperone HdeB | 1.1451 |
| **Transporter** |  |  |
| A0A0A8VB20_YERRU | Molybdenum ABC transporter periplasmic molybdenum-binding protein ModA | 0.8399 |
| A0A085U4N5_YERRU | Iron ABC transporter substrate-binding protein | 0.8563 |
| A0A085U464_YERRU | Protein TolB | 0.7475 |
| A0A085U7E4_YERRU | Transport associated protein | 1.0016 |
| A0A0A8VNM3_YERRU | Vitamin B12 transporter BtuB | 1.0079 |
| A0A085U729_YERRU | Ferrous iron transport protein A | 0.7875 |
| A0A085U8R6_YERRU | Biopolymer transporter Tol | 1.0258 |
| **Cell division protein** |  |  |
| A0A085U9F7_YERRU | Cell division protein FtsH | 0.8368 |
| A0A085U3F6_YERRU | Cell division protein FtsP | 0.8761 |
| A0A085UB58_YERRU | Cell division protein ZapA | 1.0779 |
| A0A085U3M5_YERRU | Cell division protein ZapB | 1.0840 |
| A0A085UBC3_YERRU | Cell division protein ZapD | 1.1412 |
| **Enzyme** |  |  |
| A0A0A8VFM5_YERRU | L,D-transpeptidase YcbB | 0.8060 |
| A0A085U6F1_YERRU | Uroporphyrinogen III C-methyltransferase | 0.8872 |
| A0A0A5FRU4_YERRU | Soluble lytic murein transglycosylase | 0.6312 |
| A0A085U3H5_YERRU | Arylsulfatase | 1.2959 |
| A0A085U355_YERRU | Carbonic anhydrase | 0.8350 |
| A0A085U2W0_YERRU | Acetyltransferase (Fragment) | 1.1044 |
| **Others** |  |  |
| A0A085UAP1_YERRU | 21 kDa hemolysin | 0.1232 |
| A0A085UAP8_YERRU | Putative exported protein | 1.0111 |
| A0A085U2V9_YERRU | Protein ygiW | 0.9206 |
| A0A085UAM9_YERRU | Periplasmic protein YqjC | 0.9973 |
| A0A085UAX8_YERRU | Protein yjbR | 1.1269 |
| A0A085U6W8_YERRU | YERRU Protein SlyX | 1.0190 |
| A0A085UBH1_YERRU | Protein ApaG | 0.9946 |
| A0A085U687_YERRU | Copper resistance protein | 0.9931 |
| A0A085U401_YERRU | Carbon storage regulator homolog | 1.0083 |
| A0A085U7Q1_YERRU | Heat-inducible protein | 1.0356 |
| A0A0A8V8B5_YERRU | Chromosome (Plasmid) partitioning protein ParB | 1.1009 |
| A0A085U9X2_YERRU | Chromosome partition protein MukB | 0.9852 |
| A0A085U9X1_YERRU | Chromosome partition protein MukE | 1.0946 |
| A0A0A8VDJ4_YERRU | Chromosome partitioning protein ParA | 1.0038 |
| A0A085U4I2_YERRU | Phosphate-starvation-inducible protein PsiF | 1.0193 |
| A0A085U2J8_YERRU | N-acetylneuraminate mutarotase | 1.1089 |
| A0A085U8L1_YERRU | MltA-interacting protein | 1.0361 |
